# Politics of delay hinder the implementation of EU Forest Strategy in Finland

**DOI:** 10.1007/s13280-025-02207-8

**Published:** 2025-07-10

**Authors:** Niina Pietarinen, Mireia Pecurul-Botines, Maria Brockhaus

**Affiliations:** 1 University of Helsinki, Latokartanonkaari 7, 00790 Helsinki, Finland; 2 Forest Science and Technology Centre of Catalonia (CTFC), Solsona, Spain

**Keywords:** European union, Forest policy, Policy delay, Policy transfer

## Abstract

This study analyses potential barriers to the implementation of the New EU Forest Strategy 2030 (EUFS) in Finland. When the EUFS was published in 2021, emphasising environmental outcomes and forests as carbon sinks, the timber-oriented countries were cautious of the strategy. While the EUFS is not a legally binding document, it comes with legally binding forest-related regulation. To shed light on a Member State’s response to these policy developments, policy documents and interview data was analysed through a critical institutional policy analysis lens, using policy transfer and policy delay frameworks. “All talk, little action” was the most prominent strategy in delaying implementation of the EUFS, partly legitimized by concerns that Nordic forestry expertise and needs are sidelined in the EU, and by emphasising private property rights and national sovereignty. This research demonstrates that national priorities can override international climate and biodiversity commitments, highlighting a critical gap between formal commitments and real-world practices.

**Supplementary Information:**

The online version contains supplementary material available at 10.1007/s13280-025-02207-8.

## Introduction

The focus of forest governance has broadened from wood production and biodiversity considerations to include climate change mitigation and adaptation, with carbon sequestration emerging as a central goal (Pitzén et al. 2023; Fleckenstein 2024). Simultaneously, environmental policy making has shifted from largely state-dominated, to a multi-level, shared responsibility of national and transnational actors (Arts and Leroy 2006). The New European Forest Strategy 2030 (EUFS) was published by the European Commission (EC) in 2021 as the flagship initiative of the European Green Deal, and its objective is to “set a vision and concrete actions to improve the quantity and quality of EU forests and strengthen their protection, restoration and resilience” (EC 2021). When the EUFS was published, emphasising environmental outcomes and forest carbon pools (Köhl et al. 2021), it was received by the Member States (MS) with varying reactions. Depending on MS preferences regarding aligning their policies with the EU, and their capacities to pursue such preferences, MS may follow various strategies to influence EU policy processes (Aggestam and Pülzl 2020). The traditionally timber-oriented countries, including Finland, Germany, and Austria, resisted the EUFS (Bundesministerium für Ernährung und Landwirtschaft Deutschland & Bundesministerium für Landwirtschaft, Regionen und Tourismus Österreich 2021; Ministry of Agriculture and Forestry of Finland 2021), while countries such as Belgium, Denmark, Luxembourg, the Netherlands, and Slovakia voiced support (Council of European Union 2021).

Although the EUFS is not a legally binding document, it is embedded into a wider political framework, including forest-related legislation such as the EU Taxonomy Regulation, the EU Deforestation Regulation, the Nature Restoration Regulation, the Renewable Energy Directive, as well as the European Land-Use, Land-Use Change and Forestry (LULUCF) regulation (Pecurul-Botines et al. 2023). The latter ties the MS forests into global climate mitigation efforts; consequently, national implementation of some aspects of the EUFS is required. In accordance with the European Climate Law, EU MS is required to aim for carbon neutrality by 2050. To achieve this objective, MS must reduce carbon emissions and enhance carbon removals, with the target levels for carbon balance established under the LULUCF Regulation. Finland, with comparatively high forest cover and contribution of forest industry to national GDP, faces the challenge of ongoing biodiversity loss and a collapse in the size of its forest carbon sink despite a long history of national forest legislation and programmes (Hyvärinen et al. 2019; Natural Resources Institute Finland 2022; Mönkkönen et al. 2022). Moreover, recent findings indicate that Finland is not meeting its LULUCF commitments for the first monitoring period (Soimakallio and Pihlainen 2024).

In this study, we assess the potential barriers to the implementation of the EUFS and the related regulations. The earlier works on implementation gaps in the context of environmental policy, and climate change action in particular, highlighted how discursive practices justify and legitimise in-action and delay, and how these politics of delay spread across countries and regions (Lamb et al. 2020). Consequently, in our analysis of the EUFS and the case of Finland, we pay particular attention to resistance and delays as expressed and enabled in policy and discursive practice. We analyse domestic policy priorities and the proposed measures from sectoral policy documents, supplemented with stakeholder interviews. The overarching question that this study aims to answer is how an EU MS responds to a policy framework it must formally engage with, using the case of Finland. Specifically, we ask (1) to what extent do domestic policy priorities align or conflict with the EUFS, and where are the possible mismatches, and (2) which discursive practices are employed that respond to or resist the EUFS? The research data are analysed through a critical institutional policy analysis lens (Ostrom 1992; Scott 2001), further supported by theory on policy transfer (Stone 2004) and policy delay (Karlsson and Gilek 2020; Lamb et al. 2020) to identify “discourses and practices of delayed action” (Vandenberg 2024, 122).

We argue that while Finland has formally committed to international and national forest-related regulations, forest policy actors use a selection of delay strategies to hinder the change away from business-as-usual practices, challenging policy implementation. This study addresses a gap in the forest policy literature by examining the underlying reasons—and reasoning—for delays in action following commitments to international and domestic agreements, even when there is extensive knowledge of supportive tools for working towards the intended goals. Our analysis sheds light on why, despite abundant knowledge and the urgency of climate action, countries often struggle to turn commitments into timely action. The next sections will provide background and context for the case through introduction of recent policy developments and by reviewing literature on forest policy priorities and conflicts in Finland. We then present our theoretical framework, data and methods, and research results and end with discussion and conclusion.

## Context: policy developments, priorities, and conflicts in Finland

### The new policy cycle in Finland

In the period from 2021 to 2024, Finland revised its forest-related sectoral policies to align them with its international, EU, and domestic climate obligations, initiating a new policy cycle. This process was driven by climate and environmental concerns, as well as changes in Finland’s broader operating environment, which we understand as the political and economic space in which Finland interacts with other forces. Enacting the new Climate Act (423/2022) (the Act) was a significant policy development in 2022: the Act establishes a framework for climate policy planning, monitoring, and implementation in Finland, setting ambitious national targets such as achieving carbon neutrality by 2035 and strengthening carbon sinks.

The effort to revise the forest-related policies (Table 1) was initiated under the leadership of Prime Minister Sanna Marin (2019–2023) and continued with the subsequent government led by Prime Minister Petteri Orpo (2023 onwards). Under the Act, Finland's climate policy planning system was renewed and the National Climate Change Adaptation Plan (NAP2030) and the Climate Plan for the Land Use Sector (MISU, abbreviation from “Maankäyttösektorin Ilmastosuunnitelma”) were published. In addition to legislative changes, shifts in Finland's operating environment influenced the decision to update all key forest-related policies. Key drivers of change included geopolitical risks, climate change, biodiversity loss, and shifts in consumer behaviour, all of which have implications for the forest sector (Natural Resources Institute Finland 2022). The geopolitical situation was disrupted by Russia’s invasion of Ukraine in 2022, which ended trade and wood imports from Russia, leading to an "energy crisis" in Finland, which provoked urgency to enhance energy self-sufficiency. Simultaneously, calculations by Natural Resources Institute Finland (LUKE) revealed a concerning decline in Finland’s forest carbon sink, requiring immediate policy responses in the forest sector to mitigate this trend (Natural Resources Institute Finland 2022).

**Table 1:** Policy documents included in the analysis

Document name	Acronym	Prepared by	Adopted	Time span	Description of the document (direct quote)
Framework: Carbon Neutral Finland 2035					
Climate Plan for the Land Use Sector	MISU	Ministry of Agriculture and Forestry	2023	2023–2035	The purpose of the plan is to promote the reduction of emissions from land use, forestry and agriculture, strengthening of removals by carbon sinks and adaptation to climate change, in accordance with the Sustainable Development Goals. The annual net impact that the additional measures in the land use sector aim for is at least three million tonnes carbon dioxide equivalent by 2035. The Climate Plan for the Land Use Sector contributes to the achievement of the national target of carbon neutrality by 2035 and of the climate objectives of the EU
Finland’s National Climate Change Adaptation Plan until 2030	NAP2030	Coordinated by the Ministry of Agriculture and Forestry + 9 other ministries	2024	2024–2030	The National Climate Change Adaptation Plan 2030 (NAP2030) presents a climate change risk and vulnerability assessment and sets out the vision and three aims for national adaptation work in Finland. The aims are elaborated further through ten themes for which targets and actions are specified. The Plan is part of Finland’s climate policy planning system as stated by the Climate Law
Carbon neutral Finland 2035 – National Climate and Energy Strategy	C&E	Ministry of Economic Affairs and Employment	2022	2022–2035	The National Climate and Energy Strategy outlines measures by which Finland will meet the EU’s climate commitments for 2030 and achieve the targets set in the Climate Change Act for reducing greenhouse gas emissions by 60 per cent by 2030 and being carbon neutral by 2035
Framework: Management of forest resources and forest ecosystem services					
National Forest Strategy 2035 (+ action plan for 2023–2027)	NFS2035	Ministry of Agriculture and Forestry	2022	2022–2035	The National Forest Council approved The National Forest Strategy 2035 on 14 December 2022. The process to reform the strategy was based on the rapid changes in the operating environment, both in Finland and internationally. The new strategy to 2035 considers comprehensive sustainable development in a proactive and timely manner and takes into account the role of forests in climate change mitigation and adaptation. It describes the key objectives and priorities for the development of the forest sector. The strategy has numerous interlinkages to other national and international strategies
The Finnish Bioeconomy Strategy. Sustainably towards higher value added	BE	Ministry of Economic Affairs and Employment + 6 other ministries, the Prime Minister's Office	2022	2022–2035	In July 2020, the Ministry of Economic Affairs and Employment launched a project to update the Bioeconomy Strategy. The project recognised that increasing the bioeconomy’s value added is an important focus area which is not comprehensively and holistically discussed in other strategies. The Bioeconomy Strategy extends to 2035. Its vision is “sustainably towards higher value added”. In order to promote the well-being of society, attention is paid to the holistic sustainability of the bioeconomy and the fair distribution of benefits and disadvantages
Metka (Act on the Temporary Incentive System for Forestry)	METKA	Ministry of Agriculture and Forestry	2024	2024–2029	The purpose of this act is to promote and harmonise economically, ecologically, and socially sustainable forest management and use. Pursuant to this law, measures are financed with the purpose of: 1) increase the growth of forests; 2) to secure and increase the biological diversity of forests; 3) promote water conservation in forestry; 4) curb climate change and promote adaptation of forests to climate change; 5) maintains the forestry road network
National action plan for the conservation and sustainable use of biodiversity in Finland 2013–2020	BD	Ministry of Environment	2013	2013–2020	The Strategy for the Conservation and Sustainable Use of Biodiversity in Finland for 2012–2020 is based on all of the issues covered by the CBD. It aims to promote the ecologically, economically, socially and culturally sustainable utilisation and development of biodiversity and natural resources in Finland, while safeguarding biodiversity, the vital needs of future generations, and livelihoods based on natural resources
Helmi habitats programme	HELMI	Ministry of Environment, Ministry of Agriculture and Forestry	2021	2021–2030	Helmi habitats programme aims to strengthen Finland’s biodiversity and safeguard the vital ecosystem services that nature provides for us. At the same time, the programme is working to curb climate change and promote adaptation to it
Forest Biodiversity Programme for Southern Finland	METSO	Ministry of Environment, Ministry of Agriculture and Forestry	2014	2014–2025 2025–2030	METSO aims to halt the ongoing decline in the biodiversity of forest habitats and species, and ensure that a favourable trend in forest biodiversity is established by 2025
Other domestic programmes					
A strong and committed Finland: Programme of Prime Minister Petteri Orpo’s Government	Government Programme	Finnish Government, Prime Minister’s Office	2023	2024-	The government is seeking to make Finland a strong and committed country that can withstand global storms

### The Finnish forest policy arena—Incoherence and unresolved trade-offs

Forests play a key role in both EU and Finnish climate change mitigation and adaptation strategies due to their function as carbon sinks. Finland has a long tradition of preparing forest policy strategies that aim to coordinate the various demands for forests in economically, socially, and ecologically sustainable ways. Despite these efforts, research has revealed incoherence and unresolved trade-offs in the Finnish forest domain, between and within sectoral policies, and levels (Blattert et al. 2022; Pecurul-Botines et al. 2023; Pietarinen et al. 2023; Pitzén et al. 2023). Recent research on the Finnish forest policy domain has identified a persistent emphasis on economic values in forest use, and “symbolic” commitment to climate action (Pitzén et al. 2023). Furthermore, unresolved trade-offs in forest use and knowledge disagreements have led to incoherence between forest and biodiversity policies, as argued, for example, by Pitzén et al. (2023) with a negative influence on sectoral coherence. Comparatively, on the European scale, Finland, along other MS with a nationally significant forest sector, has demonstrated limited political willingness to implement the EU's forest-related initiatives (Winkel and Sotirov 2016; Pecurul-Botines et al. 2023).

The Finnish forest sector is characterised by various discursive strategies that are used in the domain to influence international and domestic policy transfer and implementation. Sivonen and Syväterä (2023) analysed discourses in Finnish media between 2017–2018 around the EU’s LULUCF regulation. The forest industry and the government launched lobbying campaigns to influence calculation methods for LULUCF carbon sinks and to justify increased logging, actions that contrasted with their formal commitment to climate mitigation and the views of the wider scientific community. Kukkonen and Malkamäki (2024) analysed media debate on forest policy between 2015 and 2020 and found that a business-as-usual forestry coalition, as earlier observed by Harrinkari et al. (2017), defended increased logging and challenged the scientific consensus on its negative environmental impacts by emphasising technical expertise and disputing its opponents’ competence in forestry.

Given the international, EU, and domestic shifts in the forest sector’s operating environment, and the previous research done on the Finnish forest sector, it is timely to evaluate how Finland's new policy cycle addresses the diverse demands placed on its forests. This includes understanding which demands are successfully met and identifying potential shortcomings. This assessment is essential for ensuring that the revised policies are comprehensive and support Finland's international and domestic climate and environmental commitments.

## Theoretical framework

### Policy transfer

Dolowitz and Marsh (1996) introduced the concept of "policy transfer," describing it as a process, which can be voluntary, negotiated, or coercive (Dolowitz and Marsh 1996; Evans 2009), in which policies are transferred from one country to another. Policy transfer can be defined as "a process by which knowledge of policies, administrative arrangements, institutions, and ideas in one political system (past or present) is used in the development of policies, administrative arrangements, institutions and ideas in another political setting” (Dolowitz 2000, 3). Other scholars taking a constructivist perspective (McCann and Ward 2012; Stone 2017) have challenged the orthodox view of policy transfer as too simplistic for assuming policy transfer as a linear movement from point A to B. Instead, policy transfer has been defined as a nonlinear process, an assemblage—that is, a constructed and continually evolving entity—shaped by the actors involved in the process (McCann and Ward 2012).

The influence of supra-national institutions—particularly the European Union—has become an appealing area of policy transfer research (Benson and Jordan 2011). The EU facilitates the transfer of various policies across member states in multiple sectors, from environmental regulation to foreign policy (Benson and Jordan 2011). While some transfers are coercive—where a government or institution pressures others to adopt certain policies (Dolowitz and Marsh 1996)—most EU decision-making involves negotiated policy transfer, where MS try reaching consensus through dialogue (Evans 2009).

### Policy delay

Delays in policy transfer are inherent in political processes and stem from multiple and interacting sources (Varjopuro et al. 2014; Karlsson and Gilek 2020). When a policy is transferred from the EU to a MS, a "hard transfer," where the policy is adopted with minimal modifications to fit the local context, is often involved (Stone 2004). Hard transfer is often a gradual process, initiated by external pressures necessitating change, such as new legislation and policy frameworks. This process can intensify the existing barriers to policy transfer, such as path dependency and institutional constraints, making it challenging for the receiving country to align the new policy with its established systems and practices (Dolowitz and Marsh 1996; Stone 2004; Benson and Jordan 2011). The lack of a common ideology and insufficient technological, economic, bureaucratic, and political resources further complicate the process. These challenges are particularly pronounced during the implementation phase, when political, cognitive, and environmental barriers, along with domestic public opinion, can hinder progress (Evans 2009).

However, delays in implementation take place even when policy goals are democratically agreed upon and there are sufficient knowledge, technology, and economic resources to implement the policies (Karlsson and Westling 2017 in Karlsson and Gilek 2020). In the receiving country, there is typically at least a symbolic or verbal commitment, and often progress towards the policy goal, rather than a complete standstill or a refusal to implement the transferred policy (Karlsson and Gilek 2020). The literature on delays in environmental governance has aimed to understand why policy objectives or legal commitments often fail to translate into timely action, although progress may occur outside the set timeframe. These delays have been studied by researchers and called “systematic delays” (Varjopuro et al. 2014), “delay mechanisms” (Karlsson and Gilek 2020), and “discourses of climate delay” (Lamb et al. 2020). In this paper, we refer to the earlier literature under the umbrella term “politics of delay”. The use of discursive power by policy actors is central to politics of delay as it can be leveraged to delay regulatory measures (Herzog 2022; Vandenberg 2024). In line with Sivonen and Syväterä (2023), we argue that discourses can be used in rationalising the separation of actual practices from formal commitments.

Increasing our understanding of the workings of the politics of delay is necessary when trying to understand responses—or the lack thereof—to environmental policy problems, and to explain the persistence of problems such as climate change (Varjopuro et al. 2014). Such analysis will help to highlight the mechanisms at play when implementation is delayed, to increase our understanding of how to counteract delay, and to create a framework of delay strategies that allows systematic and comparative analysis (Karlsson and Gilek 2020). Lamb et al. (2020) defined 12 discourses of climate delay, grouped into those that: (1) redirect responsibility; (2) push non-transformative solutions; (3) emphasise the downsides of climate policies; or (4) surrender to climate change (Lamb et al. 2020, 1). The different delay mechanisms can exist at the same time, interact in a multidirectional manner, and build on combination of arguments (Karlsson and Gilek 2020; Lamb et al. 2020). We use the typology developed by Lamb et al. (2020) to identify the discourses that are used in the Finnish forest policy arena (Table 2).

**Table 2:** Typology of discourses of climate delay developed by Lamb et al. (2020, p.2)

1. Push non-transformative solutions	Disruptive change is not necessary
1.1 All talk, little action	Communicating ambitious climate-related goals and commitments
1.2 No sticks, just carrots	Society will only respond to supportive and voluntary policies, restrictive measures should be abandoned
1.3 Technological optimism	We should focus on future technologies
1.4 Fossil fuel solutionism	Fossil fuels are part of the solution
2. Redirect responsibility	Someone else should take action first
2.1 Individualism	Individuals and consumers are ultimately responsible for taking actions to address climate change
2.2 Whataboutism	Our carbon footprint is trivial compared to…
2.3 The "Free rider" excuse	Reducing emissions will weaken our position against…
3. Emphasise the downsides	Change will be disruptive
3.1 Appeal to social justice	Climate action will generate large costs to the society
3.2 Policy perfectionism	We should seek only perfectly-crafted solutions that are agreed with all affected parties
3.3 Appeal to well-being	Fossil fuels are required for development
4. Surrender	It's not possible to mitigate climate change
4.1 Change is impossible	Climate change mitigation and adaptation will run against current ways of life
4.2 Doomism	Any action we take is too little, too late

## Materials and methods

**Fig. 1:**
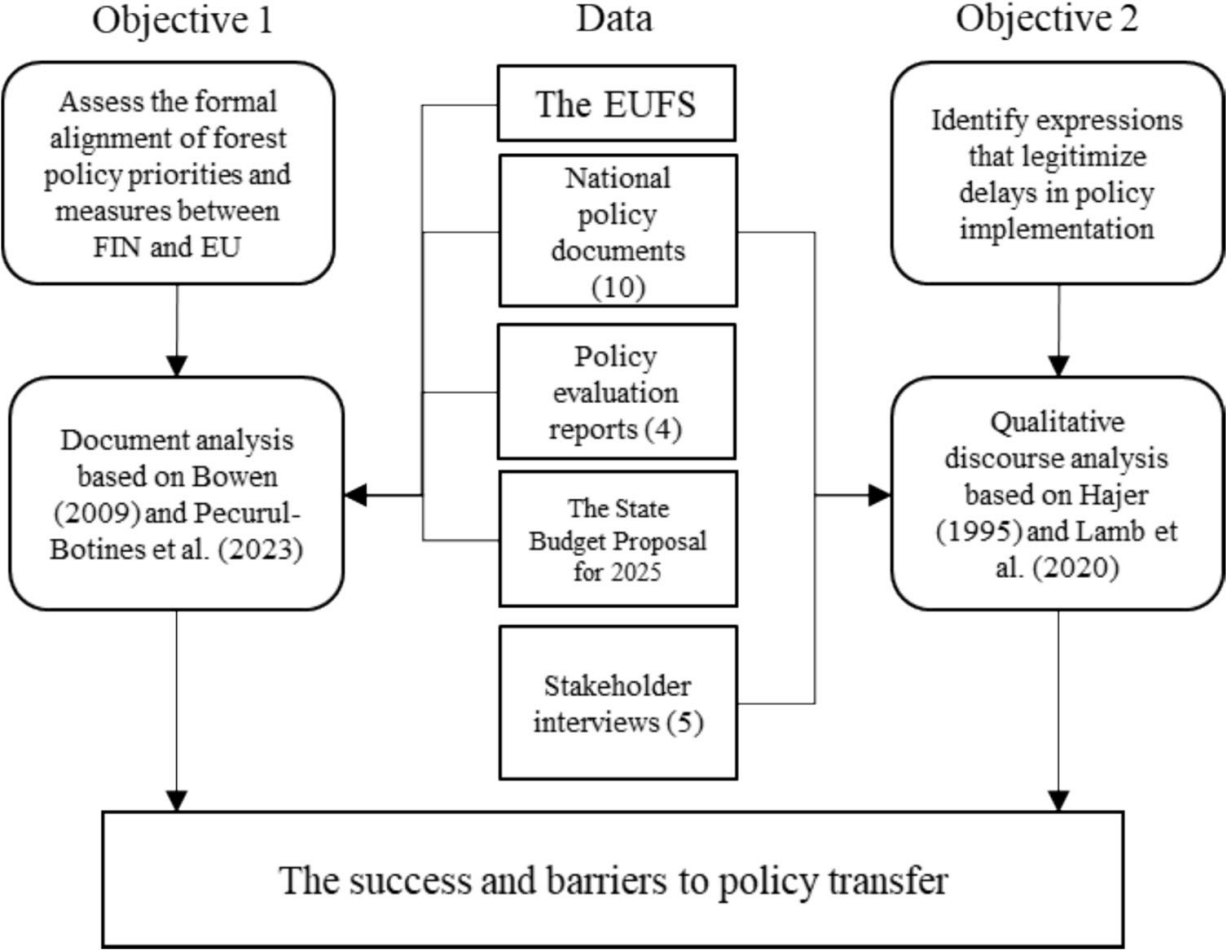
Data and methodological approach of the two parts of the analysis

This chapter outlines the data and methods used to evaluate whether and to what extent Finnish forest-related policies align with the EUFS and to identify potential discursive barriers, or delays, in implementation. The study materials consist of 10 sectoral policy documents (Table 1, Appendix 1) supplemented with five stakeholder interviews. The research has two objectives (Fig. 1): First, understanding the outcome of policy transfer by identifying the formal alignment of Finnish forest-related policy goals and measures with the EUFS through a document analysis (Bowen 2009, Pecurul-Botines et al. 2023). The second objective is to examine and identify language and argumentation that expresses or legitimises delays in policy implementation. Here, we align our critical discourse analysis with Hajer’s approach to discourse (Hajer 1995) and the typology of delay strategies developed by Lamb et al. (2020). Finland has developed several strategic plans to address climate change, enhance forest vitality, and promote sustainable use of natural resources. To capture a full understanding of the study context, the key policy documents written by public authorities from forest, biodiversity, climate change, energy, and bioeconomy sectors were examined (Table 1).

Given that the Finnish Government Programme includes forests and nature as focus areas and is responsible for funding allocation, it was included in this analysis. The document analysis was conducted by first identifying the document's overall objectives, the more specific forest-related goals, and proposed measures to reach the goals. In this study, we understand a policy measure as an instrument or action proposed by public authorities to steer, regulate, or support forest-related activities and forest management and use. Typically, policy measures are a mix of regulatory, economic, and informational instruments, such as binding regulations, subsidies, guidance, and advisory services. Sometimes a policy measure is framed vaguely, making it difficult to assess its intended effects. Then, we sought information from the surrounding context, such as the broader objective and goal. Next, we reviewed the funding that was available for each policy measure to assess the likelihood of implementation, assuming that the funded measures were prioritised. To understand allocation of funds to different forest-related policy measures, we relied on the information provided in the policy documents and the State Budget Proposal for 2025 (Ministry of Finance of Finland 2024). As climate policy and climate neutrality targets form a central part of the current policy cycle, the climate impact of each measure was also reviewed using policy evaluation reports from Laturi et al. (2022) and Laine et al. (2022), a recommendation from the Finnish Climate Change Panel (2023), and the Annual Climate Report (Ministry of Environment of Finland 2024). As complete information about funding and the climate impacts were not available for all proposed measures, the review is not systematic. However, it allows us to assess which forest-related policy measures have been prioritised through funding and which goals they support, in alignment (or not) with the goals set out in the EUFS. Moreover, this analysis was not meant to be a quantitative analysis, but a qualitative assessment of alignment and gaps, priorities, and potential conflicts. The findings were compared to policy measures put forward in the EUFS, following the typology from Pecurul-Botines et al. (2023), where a list of EUFS policy measures was developed to aid comparison between MS and EU forest policies.

Five semi-structured policy expert interviews were conducted to supplement the document analysis and to understand policy actors’ views on national forest-related priorities, conflicts, and the factors that shape those and have the potential to drive change. In accordance with the institutional policy analysis literature (Ostrom 1992; Scott 2001), we assume that actors are guided by what is deemed appropriate within their institutional context, which includes the norms, objectives, and beliefs dominant within the institution. Consequently, an action that is endorsed in one institutional context may not be supported in another. Furthermore, policy actors' political motivations and perspectives influence forest policy outcomes (Winkel and Sotirov 2016). Therefore, interviewing stakeholders who are involved in the national policy making provides insights into their motivations for pursuing different objectives. The interviewees were representatives of organisations that participate actively in forest policy making in Finland, including two government ministries, one research institute, and two national forest-related associations. The aim was to make sure a variety of organisations with diverging views and expertise on forest policy were included in the sample. The interviews were conducted either in-person or online, varied from 40 to 71 min in length, and were recorded with the participants' consent. The interview guide was developed by the second author, and all interviews were conducted and transcribed by the first author to allow thorough analysis.

The analysis was conducted by coding the policy documents and interview transcripts through the lens of the delay strategies identified by Lamb et al. (2020), providing a structured approach. The identification of language that expresses or legitimises delays in climate action in the policy documents and interview data was supported by the qualitative data analysis software ATLAS.ti, which allows the organisation and coding of textual data. During the coding process, attention was paid to any additional delay strategies that might emerge from the data. However, it was determined that the list of delay strategies developed by Lamb et al. (2020) was sufficient and no new or additional delay strategies emerged during the coding process, supporting the relevance and applicability of the existing framework.

## Results

### Formal alignment with the EUFS

This section reviews the alignment of Finnish forest-related policies with the goals of the EUFS. The EUFS aims to “deliver growing, healthy, diverse and resilient EU forests and ensure their significant contribution to our climate and biodiversity ambitions, thriving livelihoods in rural areas and beyond, and a sustainable forest bioeconomy” (EC 2021, 26). The results are divided into three themes: goals that support forests’ socio-economic functions; goals that support the protection, enlargement, and enhancement of EU’s forests; and goals that support knowledge-driven, inclusive, and coherent policy making.

#### Socio-economic functions

Sustainable timber production is a central topic in Finnish forest policy. The EUFS approach, which presents production as mutually beneficial for the transition to climate neutrality, while also supporting biodiversity and functioning as a carbon sink, aligns with claims of the Finnish forest sector of win–win outcomes as already delivered by the country’s current forestry paradigm. The Finnish forest industry uses roundwood primarily for pulp production (for short-lived products such as paper and packaging) and sawmilling (for longer-lived products), with pulpwood accounting for a larger share than logs in 2024 (36.0 million cubic meters and 25.7 million cubic meters respectively, Official Statistics of Finland 2025a, b).

The National Forest Strategy 2035 (NFS2035), the Finnish Bioeconomy Strategy (BE), the Government Programme, and the Climate Plan for the Land Use Sector (MISU) aim to promote wood-based value chains, increase the value added of forest-based bioeconomy, and produce long-lived wood products that store carbon, in alignment with the EUFS. MISU suggests measures to promote wood use in structures that maintain carbon stocks over the long term, such as construction and furniture. BE seeks to enhance the efficiency of wood construction, and the Government Programme aims to reduce regulatory barriers and introduce incentives to encourage wood construction.

Finland’s National Climate Change Adaptation Plan until 2030 (NAP2030) raises the concern that climate change can lead to potential losses of livelihoods, specifically highlighting possible unknown negative impacts for the forest sector, especially with regard to the economies of rural regions in Finland. Rural livelihoods form a critical component of Finnish forest-related policies, particularly NFS2035, BE, and the Government Programme in alignment with the EUFS. However, the approach towards rural income opportunities has caused friction between the Finnish policies and the EUFS. The NFS2035, without specifying which policy, states that EU policy measures have “neglected” (p.16) economic objectives related to wood production and the possible negative consequences for rural areas by emphasising biodiversity and forest carbon. The EUFS views timber and non-timber forest products (NTFP) as equally important economic activities, especially for rural areas. In Finland, the market for NTFP remains underdeveloped or largely unavailable. Some plans exist, however, for the development of NTFP markets in Finland: for example, NFS2035 initiates a project focused on carbon forestry in private forests, development of ecotourism, and market-based payments for ecosystem services.

Finland departs from the EUFS in entries regarding energy wood. In Finland, the delivery of energy wood has been prioritised over climate targets in the short term, due to security of supply arguments (see Sect. "Surrender—Change is impossible: “They'll make those products somewhere anyway”"). As stated in MISU (p. 72): “One of the goals [in planning the strategy] was to prepare for changing circumstances and contribute to national security of supply. This means that the measures presented in the plan and their scope will not significantly undermine the achievement of these social goals”. In 2024, 18% of all harvested roundwood, 14.1 million cubic meters, went directly to energy production (Preliminary Data provided by The Official Statistics of Finland 2025a, b). NFS2035 notes that the need for forest-based bioenergy will remain high, especially as a transitional solution. While specific sustainability safeguards for forest-based bioenergy are not established in the Finnish policies, the Climate and Energy strategy (C&E) plans to intervene in cases where biomass production is unsustainable. C&E strategy proposes increasing the level of aid in the current incentive scheme for small-diameter wood collection for energy, and for the Finnish Forest Centre to carry out a campaign to advise forest owners to collect small-diameter wood from young stands management sites (C&E, p.82). According to the EUFS, the MS should increase the share of renewable energy in their energy mix to achieve the EU's 55% emission reduction target by 2030. However, the use of bioenergy should align with carbon sink and biodiversity objectives, necessitating the strengthening of sustainability criteria for forest-based bioenergy. Moreover, the EUFS recommends that short-lived products and energy should be produced from wood that is not suitable for long-lived wood products, such as sawmill by-products, residues, and recycled materials. The EUFS recommends MS to design subsidy schemes for wood-based energy with consideration for biodiversity, yet without distorting market conditions.

#### Protecting, restoring, and enlarging EU’s forests

Finland has included climate and biodiversity targets in its forest policy, and the policies also aim to develop new ways to measure nature benefits (Ministry of Agriculture and Forestry 2024, 29). In alignment with the EUFS, Helmi Habitats Programme (HELMI), and Forest Biodiversity Programme for Southern Finland (METSO) incentivises voluntary forest conservation and restoration in private forests, and the measures put forward in MISU (e.g. preventing deforestation and using continuous cover forestry) have significant potential for climate change mitigation and adaption (Kärkkäinen et al. 2024). Furthermore, afforestation was carried out under the MISU measure, targeting treeless and unproductive lands, supporting the EUFS target to plant at least three billion more trees by 2030. The National Strategy for Biodiversity Conservation (BD) takes a cautionary stance on climate change, emphasising that the consequences of climate change for forest ecosystems are not yet well known.

The EUFS advocates for sustainable forest management to enhance climate change adaptation and forest resilience, emphasising reforestation and afforestation efforts with biodiverse, mixed-species forests, particularly those dominated by broadleaved and deciduous trees. It recommends practices such as uneven-aged and continuous cover forestry, the retention of deadwood, and minimising clearcutting due to its adverse effects on above-ground biodiversity and risks of carbon loss from tree roots and soil. These approaches are also supported by the NFS2035 and MISU. However, the existing and widely used forestry subsidy scheme for private forest owners, Act on the Temporary Incentive System for Forestry, METKA (abbreviation from “Metsätalouden kannustejärjestelmä”) predominantly supports even-aged forestry and promotes forest fertilisation to accelerate tree growth, a practice not aligned with EUFS recommendations. Contrary to EUFS, clearcutting remains the dominant forest management practice in Finland and has a strong institutional position although alternative management practices exist (see more in Sect. "Pushing non-transformative solutions—All talk, little action: “And yes, of course, we always take them seriously”").

The discussion on the criteria for the protection of old forests in Finland flared up when EUFS proposed strict protection of all primary and old-growth forests (Valtioneuvosto 2024). Environmental organisations and researchers have emphasised the need for science-based criteria to safeguard biodiversity in old-growth forests. The Finnish government proposed new protection criteria in alignment with EUFS, but the proposed criteria have come under strong criticism for not meeting the set goals (Metsälehti 2024).

#### Knowledge, inclusivity, and coherence

In Finland, forest monitoring, reporting, and data collection on forest conditions are long-established practices through the forest inventory, conducted since the 1920s (Ministry of Agriculture and Forestry: Forest Inventories). The EUFS has expressed concern over the lack of unified forest data and inadequate forest planning among the MS. Consequently, it has proposed a legislative framework for Forest Observation, Reporting, and Data Collection. The systematic process in Finland involves collecting, analysing, and reporting data on forest resources. LUKE is responsible for conducting the inventories using field plots, satellite remote sensing, and airborne laser scanning. However, it is unclear whether Finland is willing to join a unified system. The policy documents analysed include proposals for launching multiple research programmes in alignment with the EUFS goal for strong research and innovation agenda. Research is conducted, for example, on the impacts of climate action on the land use sector and on the development of carbon markets (MISU p. 88–89), climate adaptation in the forest sector (NAP2030 p.74), and on carbon stocks on forest, forest land, and forest products (NFS2035 p.30–31).

The overall research agenda aligns well with the objectives of the EUFS, with some exceptions. The current forest policy cycle in Finland has reintroduced forest fertilisation with ash and nitrogen as a method of increasing forest carbon sink and forest productivity (MISU, NFS2035, METKA)—echoing the 1960s, when fertilisation was introduced to boost forest growth (Kotilainen & Rytteri 2011). Fertilisation is not promoted under the EUFS and has a controversial image due to its potential negative impacts on water bodies, climate, and biodiversity (EC n.d.).

Finland has a longstanding tradition of participatory forest policy processes and is actively enhancing international cooperation with other forest-producing EU countries, in alignment with the EUFS recommendation for multi-stakeholder dialogue platforms. However, the quality of participation has been questioned, as further discussed in Sect. "Pushing non-transformative solutions—All talk, little action: “And yes, of course, we always take them seriously”".

### Politics of delay as implementation barriers

As demonstrated in the preceding section, policy developments have been undertaken to better align national policies with the EUFS, and with the international and national climate commitments. While the Finnish policies largely align with EUFS in goal setting, the emphasis and measures for achieving goals differ. Despite this, the progress towards achieving climate commitments remains insufficient (Soimakallio and Pihlainen 2023, Ministry of Environment 2024) and in some cases contradictory action has even been taken (Pitzén et al. 2023; Sivonen and Syväterä 2023). To understand the disparity between formal commitments and actual practices, this chapter examines discursive practices, referred to as politics of delay, that are used to postpone the implementation of formally agreed goals. The section is structured around typology from Lamb et al. (2020, Table 2) and builds on the evidence from policy documents and interviews with policy actors.

#### Pushing non-transformative solutions—All talk, little action: “And yes, of course, we always take them seriously”

The Government Programme, the NFS2035, and MISU emphasise urgency in increasing forest growth through intensified forest management and fertilisation, which is linked to national and EU-level commitments to maintain and increase the carbon sink, anticipated increases in wood use, and observed decreases in forest growth. Regardless, the annual harvesting levels are not restricted, but based on market demand. In reference to the LULUCF regulation, the Government Programme explicitly states: “The EU must not restrict the use of Finnish forests, and national interpretations must not complicate the operating environment for economic activities. The aim is to keep the harvesting opportunities agreed on with stakeholders at the present level or moderately increase them." (p.145), going against democratic commitments to climate action, and scientific consensus. This approach lies at the core of “All talk, little action” discourse, where ambitious commitments are made publicly, but the commitment does not translate into timely action. According to two interviewees, Finnish forest governance seemingly focuses on carbon sinks and biodiversity, yet it does not translate into practice. This was exemplified by an interviewee through the failure to achieve the LULUCF obligation to carbon sink in the period 2021–2025: “We are at the point where land use sector is an emission source and it tells a story of failure by forest policy to address, or even try to coherently reach the EU commitment that was set in 2018”. In contrast, another interviewee stated that the main drivers for updating the NFS2035 were the ambitious climate commitments.

Indeed, there have been significant policy developments towards climate change mitigation and adaptation, namely through MISU, NAP2030, NFS2035, and HELMI, bearing great potential to mitigate climate change in alignment with the EUFS. However, the funding mechanisms have not been established to support mitigation and adaptation measures, delaying their implementation (see also Ministry of Environment 2024). MISU has not received funding, despite being the key policy for achieving climate neutrality in the land use sector. Additionally, following its publication, it was found that the strategy was based on an overly optimistic carbon baseline scenario (Silfver et al. 2004). The primary funding mechanism for subsidising forestry work in private forests, METKA, predominantly supports practices that favour wood production and even-aged forestry (Viitala et al. 2022). For the 2025–2027 period, the budget allocated for forest management subsidies via the METKA system is approximately 36 million euros per year (Ministry of Finance of Finland 2024). In contrast, funding for the nature conservation and restoration programmes, METSO and HELMI, is set at around 7 million euros per year (Ministry of Finance of Finland 2024). The Government Programme (p.168) proposes to use the METSO funding for forest management backlogs and fertilisation in private forests, although it is a funding scheme dedicated to forest conservation in privately owned forests. Despite the reform of METKA in 2024, an interviewee noted that the funding continues to prioritise wood production. The interviewee was critical towards announcements that METKA aligns with climate goals in the land use sector when no actual changes in funding priorities were made. National Audit Office of Finland (2023) concluded that the types of activities subsidised through METKA and funding priorities have remained largely unchanged over the past 60 years.

Finnish citizens have started to demand changes in how forests are used: 75% of the respondents of the 2025 Climate Barometer wanted more attention to be paid to preserving carbon sinks in forestry practices (Ministry of Agriculture and Forestry, Ministry of Transport and Communications and Ministry of the Environment 2025). To align the various demands for forest use, the NFS2035 was prepared in a participatory policy drafting process, inviting stakeholders to join. However, environmental groups withdrew from the process due to disagreements over the strategy's objectives. One interviewee highlighted participation as crucial for conflict resolution in the forest sector but added that the environmental groups left the process because “they did not believe in it”. Another interviewee, who had participated in the preparation of two national forest programmes in Finland, said that their organisation decided to reduce their involvement as they felt that their input was not valued. All interviewees further observed that the two ministries responsible for forest governance, the Ministry of Agriculture and Forestry (MAF) and the Ministry of Environment (ME), lack dialogue, which undermines not only national resource use but also Finland’s position in the EU. One interviewee felt that the two ministries were not even willing to cooperate, while another criticised the public disputes over forest resource use between the ministries. We view the persistent gap between forest policy direction and stakeholder demands for change as a key element of the “All talk, little action” discourse.

Finally, Finnish extensive forest knowledge is frequently highlighted in both policy documents and by two interviewees to justify national sovereignty in forest use and to put the spotlight on the EU’s perceived incompetence in the forest domain. We consider this one aspect of the “All talk, little action” discourse, as the extensive knowledge seems to be harnessed mainly to support the industrial use of forests, and in finding the minimum level for sustainability while maintaining market access (Kuuluvainen et al. 2019).

#### Pushing non-transformative solutions—No sticks, just carrots: “But then it brings certain regulation to Finland”

According to an interviewee, and reflected in the Government Programme, the NFS2035, and METKA, timely forest management is the best solution to the climate and biodiversity crisis, as opposed to restricting forest use due to climate considerations. Overall, the market is viewed as the most effective mechanism for guiding forest use and determining harvest levels. This forms the core of the “No sticks, just carrots” discourse, which emphasises the ineffectiveness of regulation. The Government Programme seeks to strengthen the role of forest owners and private property rights while advocating for EU policies that do not restrict the supply of wood for industrial use. EU regulation was described by one interviewee as “forceful and threatening” and, according to the NFS2035 (p.14), it is increasingly challenging to reconcile EU regulation with national interests. The Government Programme emphasises: "Finland’s Forest Policy will be kept in our own hands" (p. 144–145). This approach represents a departure from the previous government, which aimed to align the forest-related policies with evolving EU requirements and the national carbon neutrality target. One interviewee in turn criticised deregulation using an example about the updating of the national Forest Act in 2014 that provided flexibility for forest owners, resulting in minimal regulation and virtually no sanctions for forest offences.

#### Redirecting responsibility—Individualism: “No one is forced to either sell or not sell wood”

As noted earlier, the Government Programme has taken a firm position against the influence of the EU in the forest sector. While it has committed to achieving both national and EU carbon neutrality targets, the programme explicitly states that "carbon sinks in Finnish forests cannot serve as a means to offset emissions produced by other EU Member States" (pp. 144–145), redirecting the responsibility to individual member states instead of joining collective action to mitigate climate change.

All five interviewees agreed that disagreement between harvesting levels and other forest uses is the major forest-related conflict in Finland. However, two interviewees emphasised that the decision to sell timber is made by individual forest owners, stating that "no one is forced to either sell or not sell wood", redirecting responsibility about harvest levels to individual forest owners and market demand. This perspective overlooks mechanisms highlighted in studies such as Takala et al. (2023), which indicate that the services and subsidies for Finnish forest owners predominantly support timber production in even-aged forests. One interviewee stated that despite the prominent discussions about forest owners' autonomy and the diversity among the 600 000 private forest owners in Finland, the prevailing forestry model across the country promotes unified industrial, even-aged forestry. Individualism was also employed as a discursive strategy when the criteria for protecting old-growth forests were drawn up in Finland in response to the EUFS proposal to safeguard the EU's remaining primary and old-growth forests. The criteria adopted, contrary to scientific evidence, were set so strictly that any forests in Southern Finland would barely qualify for protection. This decision was defended by the then Minister of Climate and the Environment, who stated: "The criteria concerns 600 000 forest owners. It is about the protection of property" (YLE 2024).

#### Emphasise the downsides–Appeal to social justice: “The outcome could be bad for the climate”

The Government Programme, the NFS2035, BE, and MISU emphasise the downsides of climate action by claiming forests’ socio-economic significance for Finns and the potential disruption brought by climate action. While climate denialism is not common, the central position of the forest sector for Finnish well-being through employment and cultural significance is often brought up. One interviewee speculated that leaving forests unmanaged due to climate reasons could in fact have negative consequences for the climate and the Finnish people. They argued that forest management and use enhance climate resilience through forest growth and health, and by providing alternatives to fossil fuels, a view also put forward in the policy documents. Two interviewees in turn criticised the exclusive policy emphasis on timber production, arguing that it overlooks, and sometimes conflicts with, other valuable socio-cultural benefits of forests, such as recreation and mushroom and berry picking, which are popular activities among Finns. According to three interviewees, NTFP have so far not received adequate attention in forest policy, with the markets and instruments predominantly centred around timber sales and the issuance of fishing and hunting licenses. This is despite the potential for developing a variety of other activities related to non-timber products and the ecotourism sector.

#### Surrender—Change is impossible: “They'll make those products somewhere anyway”

A perceived barrier to reducing harvest levels in Finland is the anticipated increase in the demand for forest products. Consequently, none of the analysed policy documents propose lowering the harvest levels. One interviewee argued that forest products would be produced somewhere regardless, and thus, regulatory or policy barriers should not disrupt market functioning or impose disadvantages on the Finnish forest industry.

A major theme throughout the policy documents analysed is the geopolitical situation as a reason to increase forest use in the short term. The end of wood imports from Russia has caused prioritisation of energy security and supply, which is visible in the Government Programme, the C&E, the NFS2035, MISU, and BE, which all promote energy wood collection. Interestingly, wood-based energy forms a significant part of Finland’s climate policy framework, in contrast with the EUFS, which aims to limit the use of energy wood. The C&E strategy outlines measures by which Finland can achieve both the EU’s climate commitments for 2030 and the national carbon neutrality target for 2030 and support the strengthening of the carbon sink of the land use sector. However, in contrast to these goals, the forest-related measures focus solely on the security of supply of wood for energy production and for the forest industry. For example, securing the forest chips supply received a notable allocation of 40 million € in the state budget in 2022. To secure the energy wood value chain, the C&E strategy proposes harmonising and streamlining environmental permit processes to prevent delays in establishing new biomass terminals that function as storage and processing spaces for energy wood.

## Discussion

### Barriers to the policy transfer

This research examined Finnish forest policy alignment with the New EU Forest Strategy 2030 and possible discursive barriers for implementation. We identified that both international and national developments and legislation on climate and biodiversity targets have initiated changes in Finnish forest policy, incorporating new policy goals through hard transfer. However, in alignment with theory (Stone 2004; Benson and Johnson 2011), the adoption of new practices is slow due to path dependency, perception of poor institutional alignment, and resistance within national structures.

The current approach to forest policy in Finland reflects a pattern of adopting regulation through hard transfer while delaying substantial transformative change. This type of transfer is typically conducted by politicians and government officials and follows a path-dependent, incremental, and inherently slow process (Stone 2004). As a result, the new policy agenda from the EU is framed in Finland as complementary to traditional forestry objectives, maintaining continuity rather than pushing for a paradigm shift (Pietarinen et al. 2023; Pitzén et al. 2023). This continuity is evident in the emphasis on active forest management that enhances carbon sinks and mitigates climate change, and wood products as substitutes for fossil-based products. Despite the substantial evidence for ongoing biodiversity loss in the Finnish forests, there remains an acceptance that current forest management practices support biodiversity (Takala et al. 2023).

At present, climate mitigation has arguably received more attention than biodiversity, with the prevailing discourse now suggesting that forest management contributes to climate change mitigation. This narrative remains controversial as studies (Skytt et al. 2021; Schulte et al. 2022; Soimakallio et al. 2022) suggest that reducing harvest levels to increase the forest carbon sink outperforms the climate benefit from substitution for extended time periods. The enduring narrative that traditional forest management addresses evolving challenges hence reflects significant path dependency in forestry knowledge systems and persisting institutional arrangements in the forest sector.

The primary policy focus in Finland remains on creating favourable conditions for wood production, reinforcing existing economic priorities, and forest business as usual. Policies rely predominantly on voluntary, non-binding instruments and emphasise the property rights of family forest owners, which limits the implementation of legally binding commitments to environmental and climate goals. In Finland, private forest owners are typically referred to as family forest owners, highlighting a cultural and generational tie to one’s forest. However, the autonomy of family forest owners is somewhat limited due to the policy framework, which strongly encourages wood production within a unified system. This system is supported by various subsidies and services, creating an environment where forest owners, despite having the freedom to make independent decisions, are strategically guided towards fulfilling the demands of the forest industry.

### Discursive strategies and delays

In this study, we adopted a constructivist perspective on policy analysis. It conceptualises policy as an assemblage that is constantly evolving and shaped and interpreted by diverse actors. This approach enabled our analysis to extend beyond the evaluation of formal alignment of policy goals and measures. By examining the details that influence policy direction beyond the formal policy processes, we can reveal the discursive strategies employed to delay action within the forest sector. Discourses facilitate action by shaping our collective understanding of the world and can be deliberately constructed to obstruct alternative interpretations and actions (Benjaminsen and Svarstad 2010), to avoid responsibility from acting (Lamb et al. 2020), and to be used as “rhetorical tools to delay action” (Vandenberg 2024, p. 131). Our findings reinforce and add to the earlier policy delay literature in explaining systemic failures to achieve environmental goals, supporting their applicability beyond this case study. In our case, the EU’s call for MS to adopt a new forestry paradigm has provoked resistance towards the EUFS, leading to delays.

### Implications

The Finnish forest policy approach is supported by a longstanding political-business coalition that has traditionally shaped forest policy and maintained a hegemonic influence over policy direction (Korhonen et al. 2018; Holz 2023; Lonkila et al. 2025). A lack of shared ideology between stakeholder groups and the limited inclusion of diverse stakeholders in policy making has restricted “soft transfer” (Stone 2004), the dissemination of norms, ideas, and knowledge from EU to Finland. In contrast with hard transfer, soft transfer is less restrained by institutional path dependency, instead emphasising rational, knowledge-driven policy processes that can be initiated by new knowledge, uncertainty, or crises (Stone 2004). In the Finnish case, environmental groups withdrew from the participatory forest policy process as a signal of dissatisfaction towards the narrow scope of forest policy. This dynamic highlights the need for more inclusive policy making to achieve meaningful progress in forest policy. We acknowledge that while engaging in soft transfer alone does not guarantee the adoption of the new practices required to implement a policy, it often provides the necessary foundation for policy knowledge that can eventually lead to practical implementation (Stone 2004).

Our results highlight a lack in the diversity of voices that are heard in policy making—we suggest involving Finnish society at large in shaping the visions of future forests beyond the traditional forestry paradigm. More diverse subsidies and services should be developed for forest owners, to accommodate various motivations and management objectives beyond even-aged forestry. The success of the internationally recognised METSO programme, which incentivises conservation efforts by compensating forest owners for preserving biodiversity-rich areas, serves as an example.

EU’s forest-related governance framework has faced criticism, with research highlighting internal incoherences (Köhl et al. 2021). We acknowledge that the EUFS and its accompanying regulations are not flawless forest governance frameworks. Nonetheless, given the extensive scientific knowledge on climate and biodiversity crises, and various international commitments to tackle those, it is essential to examine how climate and biodiversity-related objectives are being pursued in MS. "Policy perfectionism" (see Lamb et al. 2020) should not serve as an excuse to avoid working towards these goals. How these new legal developments will impact forest management is worth further research. Nevertheless, MS should continue to engage in dialogue and knowledge exchange and encourage a flow of ideas to address transnational challenges such as climate change and biodiversity loss.

## Conclusion

Our results highlight that the alignment of policy measures and discursive practices indicates only a very limited transformation in the Finnish forest sector. We argue that while formally committing to various national and international forest, climate, and biodiversity agreements and regulations, policy makers have employed the “All talk, little action”, “No sticks just carrots”, “Redirect responsibility to individuals”, and “Surrender” strategies to delay a change from business-as-usual. Moreover, the funding priorities in the current policy cycle indicate an ongoing emphasis on even-aged forestry and the production of short-lived wood products. We find a symbolic commitment to environmental and climate action, while policy actors redirect responsibility to individual forest owners and free markets without acknowledging the institutional mechanisms that uphold the traditional forest management paradigm. This can hinder the work towards formally accepted climate and biodiversity goals.

The politics of delay in the Finnish forest sector are likely not unique to the country or the specific sector but might be shared across and beyond MS in the wider land-use sector. Taking the concerns over climate change and biodiversity loss seriously would require stronger instruments, political ambition, and a sense of urgency to ensure that these commitments are not sidelined in policy implementation, in Finland and elsewhere.

## Supplementary Information

Below is the link to the electronic supplementary material.

Supplementary file1 (PDF 125 kb)
