# Supplementary material for: Politics of delay hinder the implementation of EU Forest Strategy in Finland
Source: Ambio. 2025 Jul 10;54(12):2154–69. doi: 10.1007/s13280-025-02207-8 (PMC12569296; doi:10.1007/s13280-025-02207-8)
Supplement: Supplementary file 1 — Supplementary file1 (PDF 125 kb) [file 13280_2025_2207_MOESM1_ESM.pdf]

Supplementary Information: This Supplementary Information has not been peer reviewed.

Title: Politics of delay hinder the implementation of EU Forest Strategy in Finland

Journal: Ambio

Authors: Niina Pietarinen, Mireia Pecurul-Botines, Maria Brockhaus

E-mail of corresponding author: niina.pietarinen@helsinki.fi

Supplementary information

Appendix S1 The analysed policy documents

Act on the Temporary Incentive System for Forestry (METKA) 71/2023. Finlex. Retrieved 20 December, 2024, from https://finlex.fi/fi/laki/ajantasa/2023/20230071 (Web material)

Finnish Government. 2022. The Finnish Bioeconomy Strategy. Sustainably towards higher value added. Publications of the Finnish Government 2022:5. http://urn.fi/URN:ISBN:978-952-383-579-5

Finnish Government. 2023. A strong and committed Finland - Programme of Prime Minister Petteri Orpo's Government 20 June 2023. Publications of the Finnish Government 2023:60. Retrieved 20 December, 2024, from https://julkaisut.valtioneuvosto.fi/bitstream/handle/10024/165042/Paaministeri-Petteri-Orpon-hallituksen-ohjelma20062023.pdf?sequence=1&isAllowed=y (Web material)

Finnish Government. 2024. Government Report on Finland's National Climate Change Adaptation Plan until 2030 Wellbeing, Safety and Security in a Changing Climate. Publications of the Finnish Government 2024:11. https://urn.fi/URN:ISBN:978-952-383-814-7

Gummerus-Rautiainen, P., Alanen, A., Eisto, K., Ilmonen, J., Keskinen, H-L., Krüger, H., Matveinen, K., Svensberg, M., Rintala, T., Raatikainen, R., Ryömä, R., Siitonen, J. 2021. Helmi-elinympäristöohjelma 2021–2030: Valtioneuvoston periaatepäätös. Valtioneuvoston julkaisuja 2021:83. http://urn.fi/URN:ISBN:978-952-383-899-4

Ministry of Agriculture and Forestry. Forest Biodiversity Programme for Southern Finland (METSO) 2014–2025. Retrieved 20 December 2024 from https://mmm.fi/metso-ohjelma (Web material)

Ministry of Agriculture and Forestry. 2023a. Government Report on the Climate Plan for the Land Use Sector. Publications of the Ministry of Agriculture and Forestry 2023:12. http://urn.fi/URN:ISBN:978-952-366-592-7

Ministry of Agriculture and Forestry. 2023b. The National Forest Strategy 2035. Publications of the Ministry of Agriculture and Forestry 2023:24. http://urn.fi/URN:ISBN:978-952-366-748-8

Ministry of Agriculture and Forestry. 2024. National Forest Strategy 2035. Action plan 2023–2027. Retrieved 11 December, 2024, from https://mmm.fi/documents/1410837/110695773/KMS2035_tps_vahvistettu+11.6.2024.pdf/df7c90cc

-754f-9999-c192b36fa55fba36/KMS2035_tps_vahvistettu+11.6.2024.pdf?t=1718117097201 (Web material)

Ministry of Economic Affairs and Employment. 2022. Carbon neutral Finland 2035 – national climate and energy strategy. Publications of the Ministry of Economic Affairs and Employment 2022:55. https://urn.fi/URN:ISBN:978-952327-843-1

Ministry of Environment. 2013. Saving Nature for People National action plan for the conservation and sustainable use of biodiversity in Finland 2013–2020. Retrieved 20 December, 2024, from https://ym.fi/documents/1410903/38439968/Luonnonpuolesta---ihmisen-hyvaksi.-Suomen-luonnon-monimuotoisuuden-suojelun-ja-kestavan-kayton-toimintaohjelma-2013%C3%A2%E2%82%AC%E2%80%9C2020-A1006DC3_DDD2_4710_AFD4_C0F29D96C110-31786.pdf/4b50b3a3-9301-9912-7dab-6b5481d4d573/Luonnon-puolesta---ihmisen-hyvaksi.-Suomen-luonnonmonimuotoisuuden-suojelun-ja-kestavan-kayton-toimintaohjelma-2013%C3%A2%E2%82%AC%E2%80%9C2020-A1006DC3_DDD2_4710_AFD4_C0F29D96C110-31786.pdf?t=1603260012095 (Web material)
